# Hyaluronic Acid Functionalization with Jeffamine^®^ M2005: A Comparison of the Thermo-Responsiveness Properties of the Hydrogel Obtained through Two Different Synthesis Routes

**DOI:** 10.3390/gels7030088

**Published:** 2021-07-09

**Authors:** Mathieu Madau, Didier Le Cerf, Virginie Dulong, Luc Picton

**Affiliations:** Laboratoire Polymères Biopolymères Surfaces (PBS), Normandie University, UNIROUEN, INSA Rouen, CNRS, 76000 Rouen, France; mathieu.madau@univ-rouen.fr (M.M.); virginie.dulong@univ-rouen.fr (V.D.); luc.picton@univ-rouen.fr (L.P.)

**Keywords:** hyaluronic acid, hydrogel, thermo-responsive, Jeffamine^®^ M2005

## Abstract

Hyaluronic acid (HA) of different molar masses (respectively 38,000, 140,000 and 1,200,000 g.mol^−1^) have been functionalized with a commercial poly(etheramine), Jeffamine^®^ M2005, in order to devise physical thermo-responsive hydrogels. Two routes have been studied, involving the use of either water for the first one or of N,N′-Dimethylformamide (DMF), a polar aprotic solvent, for the second one. In the case of the water route, the reaction was performed using a mixture of N-(3-Dimethylaminopropyl)-N′-ethylcarbodiimide (EDC) and N-hydroxysuccinimide (NHS) as coupling reagents. The reaction was optimized while making sure no free M2005 remained in the final material, leading to M2005 grafting degrees of about 4%, which enabled the formation of hydrogels by increasing the temperature. In the case of the organic solvent route, propylphosphonic anhydride T3P^®^ was used as a coupling reagent in DMF, resulting in a M2005 grafting degree of around 8% with better thermo-responsive properties of HA-g-M2005 compared to those obtained when the reaction was performed in water. However, the reaction systematically led to covalent cross-linking in the case of the HA, with the highest starting molar masses resulting in a very different rheological behaviour and with higher gel strength retaining thermo-responsive behaviour but being only poorly soluble in water.

## 1. Introduction

Hydrogels are soft materials made of a three-dimensional hydrophilic polymeric network able to swell in water [1,2]. They are increasingly studied in order to design complex materials for applications in the biomedical field, with applications in wound dressing, drug delivery, scaffolds for tissue engineering or magnetic hydrogels [3,4,5]. Hydrogels are divided into two categories: physical and chemical hydrogels. Physical hydrogels have their cross-links made of dynamic non-covalent bonds, such as hydrogen bonds [4], ionic cross-linking [6] or Van der Walls interaction [7]. These cross-links are reversible, and their association or dissociation dynamics are dependent on the physical and chemical environment of the material, such as the temperature [8], the pH [7], the saline environment [6], etc. Oppositely, chemical hydrogels are made through the formation of covalent cross-linking. Such cross-linking leads to stronger structures compared to physical hydrogels; however, they are not reversible and are therefore not as versatile as physical hydrogels. Devising hydrogels for biomedical applications requires of course to use biocompatible raw materials, with biotic materials being the preferred option [9].

Hyaluronic acid is a glycosaminoglycan found within tissues of vertebrates and some bacteria as a major component of the extracellular matrix and of connective tissues such as the synovial fluid, where it helps lubricating articulations or umbilical cord [10,11]. It is a linear polysaccharide made of glucuronic acid units alternating with N-acetylglucosamine units [12]. Among glycosaminoglycans, it is the only non-sulfated one and is not bound to a protein [13]. 

HA is very hydrophilic [10] and though it is already used for its viscoelastic properties in the cosmetic industry [13,14], it needs to be functionalized to acquire hydrogel properties. In particular, grafting a thermo-responsive moiety displaying a lower critical solution temperature (LCST) will result in a material able to undergo a sol-gel transition by raising the temperature. Some polymers do indeed display a LCST, which is the minimum temperature above which they will go from a soluble state in a given solvent (mostly water in the studied cases) to an insoluble state. It has to be stressed that the transition temperature will change according to the polymer concentration in the solvent, the LCST being the minimum temperature above which the polymer will undertake a transition. In general, the transition temperature is referred to as a cloud point [15]. There are many polymers displaying a LCST, most of them synthetic, like poly(N-isopropylacrylamide) PNIPAM, the one that is most studied, which is due to its LCST being around 32 °C in water, which is perfect for biomedical application [16]. Moreover, its transition temperature is not very dependent on the polymer concentration compared to other systems [16]. Other examples of thermo-responsive polymers are poly(N-vinylcaprolactam) [16], polyoxamers made of poly(ethylene oxide)-b-poly(propylene oxide)-b-poly(ethylene oxide) block copolymers [8], and hydroxypropyl cellulose (obtained through the functionalization of a natural material) [17]. 

HA has already been functionalized with polymers displaying a LCST. For instance, PNIPAM has been grafted either on HA using either “grafting from” [18] or “grafting to” approaches [19,20]. Another polymer, Jeffamine^®^ M2005, a poly(etheramine), with a LCST of 14°C in water, is being increasingly used as a functional group. It has a block copolymer structure consisting of 6 units of ethylene oxide EO and 29 units of propylene oxide PO ended with an amine at the PPO block end, a functionality that makes this polymer quite suitable for grafting on other polymeric structures, especially the ones displaying carboxylic acid moieties through the formation of an amide bond. Therefore, Jeffamine^®^ M2005 has first been grafted on carboxymethylpullulan by Dulong et al. [8] before the reaction was adapted to HA by Eglin et al. [20] and more recently by Trojer et al. (who also did the reaction on carboxymethylcellulose) [21]. Eglin et al. functionalized HA with both Jeffamine^®^ M2005 and M600 (with a different composition than M2005, the chain being notably much shorter), using the method they developed to graft amino terminated PNIPAM to HA in DMSO with carbonyl diimidazole as a coupling reagent. However, their HA-g-M2005 did not display any thermo-responsive properties and their HA-g-M600 gave only very limited increase of storage modulus G′ upon heating, contrary to their HA-g-PNIPAM, for which they had far more interesting results that were subsequently used by this group for their following studies [22,23]. In the case of Trojer et al., they did the coupling reaction using the well documented procedure involving N-(3-Dimethylaminopropyl)-N′-ethylcarbodiimide (EDC) in conjunction with N-hydroxysuccinimide (NHS) as the coupling reagent in water. Their resulting materials were responsive to temperature; however, their rheological studies did not include oscillation measurements, which would have helped to definitely confirm the presence of a gelation point by comparing the evolution of the storage modulus G′ and the loss modulus G″ during a temperature ramp.

**Scheme 1:**
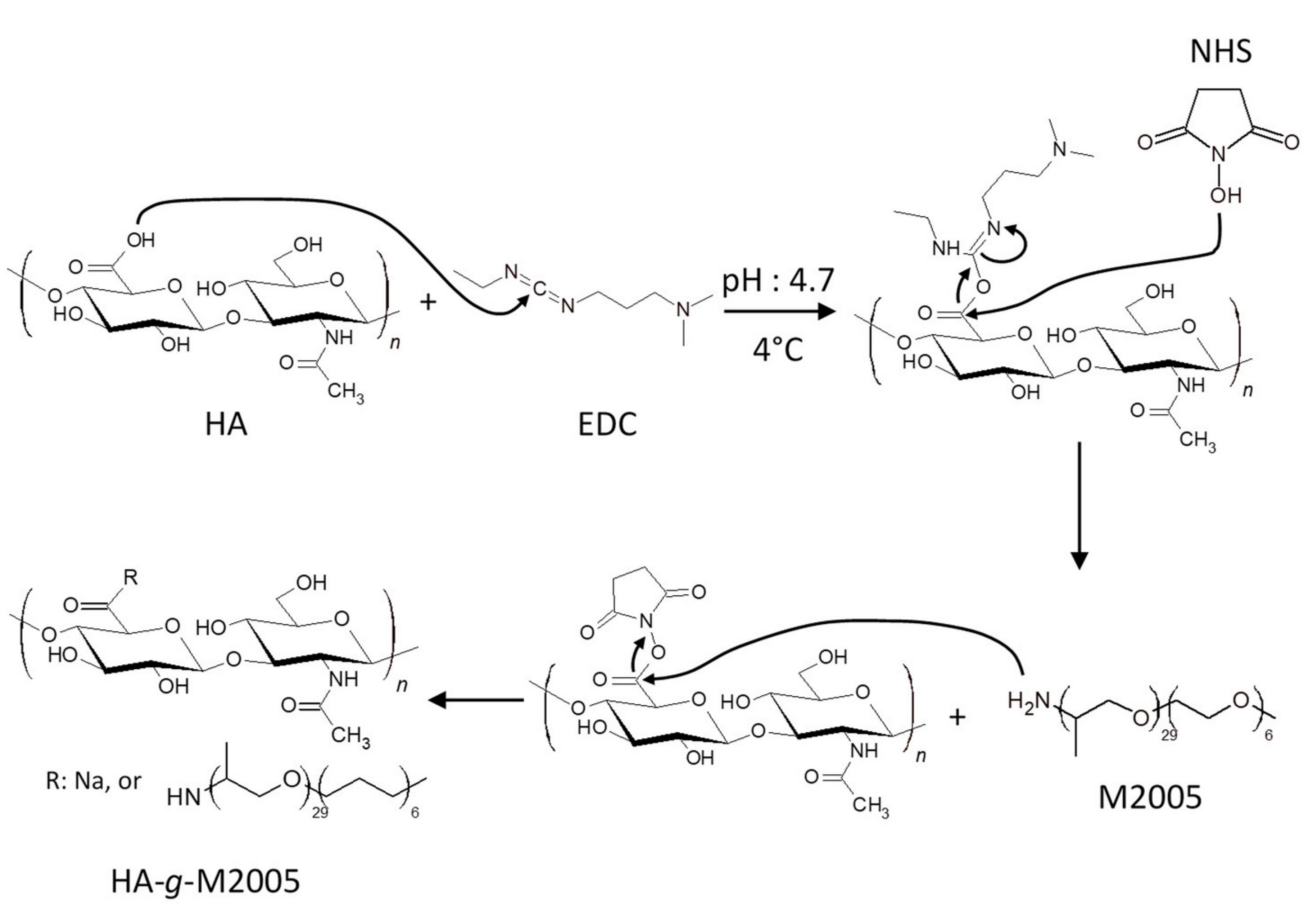
Mechanism of the grafting of M2005 to HA (water route).

Here, we are going to adapt to HA the methodology from Dulong et al., developed for grafting Jeffamine^®^ M2005 to carboxymethylpullulan in water using EDC/NHS as the coupling reagent (Scheme 1).

**Scheme 2:**
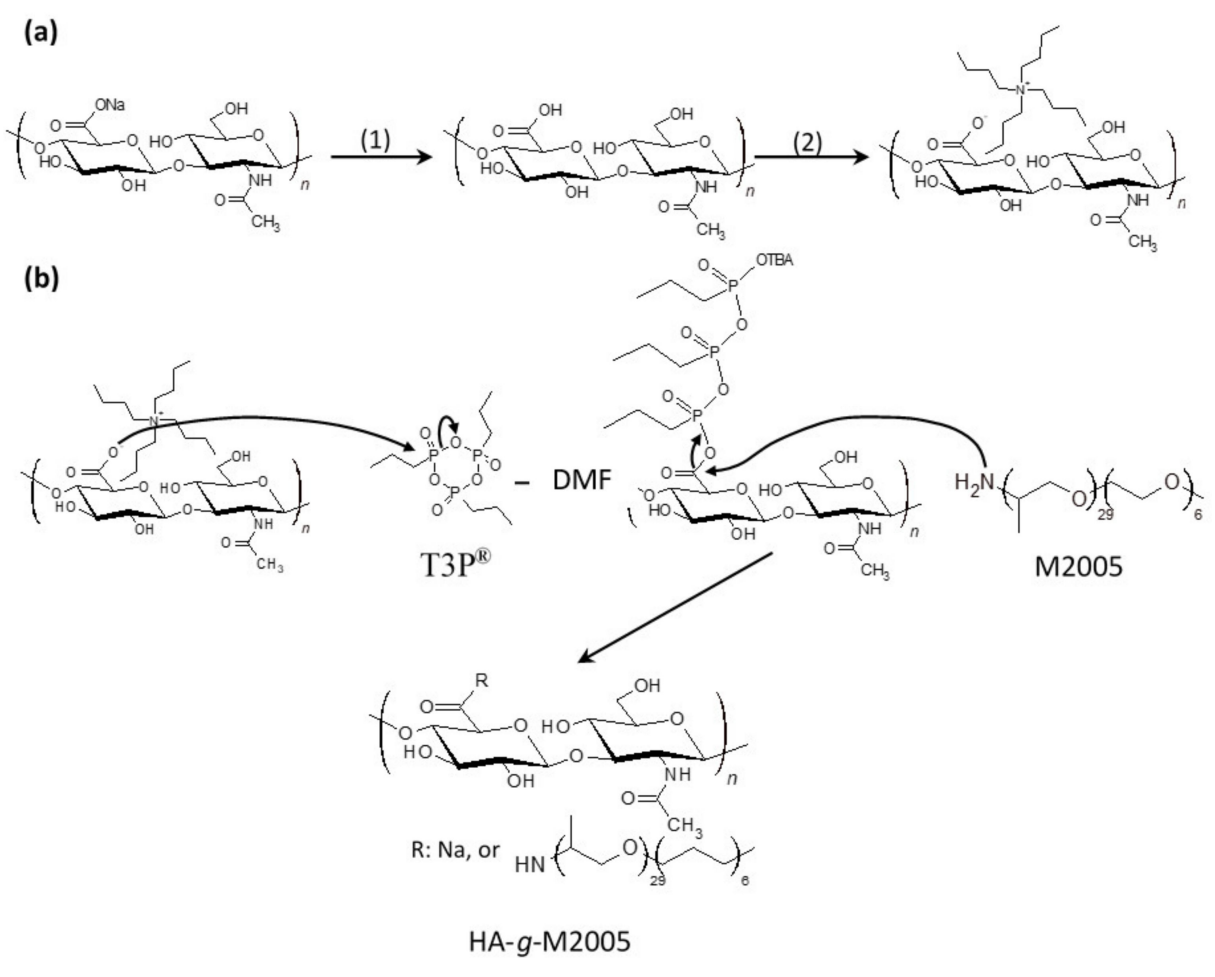
Proposed mechanism of the grafting on M2005 to HA (organic solvent route). (**a**) Conversion of Na-HA to its TBA salt through two steps: (1) acidification with a DOWEX cationic ion exchange resin overnight (the resin is then filtered off the solution) and (2) neutralization of the resulting hyaluronic acid with TBAOH. (**b**) Activation of TBA-HA with T3P^®^ followed with nucleophilic substitution with M2005, resulting in HA-g-M2005.

This protocol, in particular, puts a strong emphasis on sample purification, with short dialysis steps in acidic and alkaline media to efficiently remove the EDC (which would otherwise be attached to HA as a N-acylurea as described by Kuo et al. [24]) as well as longer dialysis steps in a mixture of ethanol and water and washing of the final material in acetone to remove unreacted M2005, which was found out to be difficult to remove using only dialysis. As the obtained degrees of substitution (DS) were low, with values below 5%, we have adapted a protocol developed by Schleeh et al. who grafted simple aliphatic amines to alginate, another polysaccharide with carboxylic acid moieties, using the coupling reagent propylphosphonic anhydride T3P^®^ in N,N′-Dimethylformamide (DMF), for which they reported DS of around 100% [25] (Scheme 2). The structure of the modified HAs has been determined through FTIR, NMR and SEC/MALS, while its thermo-gelling properties have been assessed through rheology.

## 2. Results and Discussion

### 2.1. Synthesis of HA-g_(EDC)_-M2005 in Water

M2005 was grafted to HA_1200_, HA_140_ and HA_38_ using EDC/NHS as a coupling reagent for 24 h at 4 °C, the mechanism is detailed in Scheme 1. The reaction was done under mild acidic conditions and at 4 °C in order to have M2005 in the sol state [8]. The reaction of the HA carboxylic acid moiety led to the formation of an activated O-Acylurea intermediate (Scheme 1), which can rearrange itself into an unreactive N-Acylurea. It has been reported the use of NHS in catalytic quantities helps prevent this by inducing the formation of a second activated intermediate which is more stable. Both intermediates will eventually lead to the formation of an amide bond with M2005. In order to fully remove EDC and unreacted M2005, extensive purification steps had to be undertaken, using first dialysis against various solutions of NaOH, HCl or water (see Section 4.2.1 for details). These steps are needed to make sure no EDC remains in the final material. In order to fully remove unreacted M2005, dialysis against a mixture of ethanol and water (ratios 33/67 *v*/*v*) was performed, followed by dialysis against water, and the resulting material received after freeze-drying was then washed several times in acetone until no more M2005 grafting degree variation was observed through NMR (Figure S1). The resulting functionalized HAs using this route were coded as follow: HA_Mn(kg.mol_^−1^)-g_(EDC)_-M2005-DS%. 

**Figure 1:**
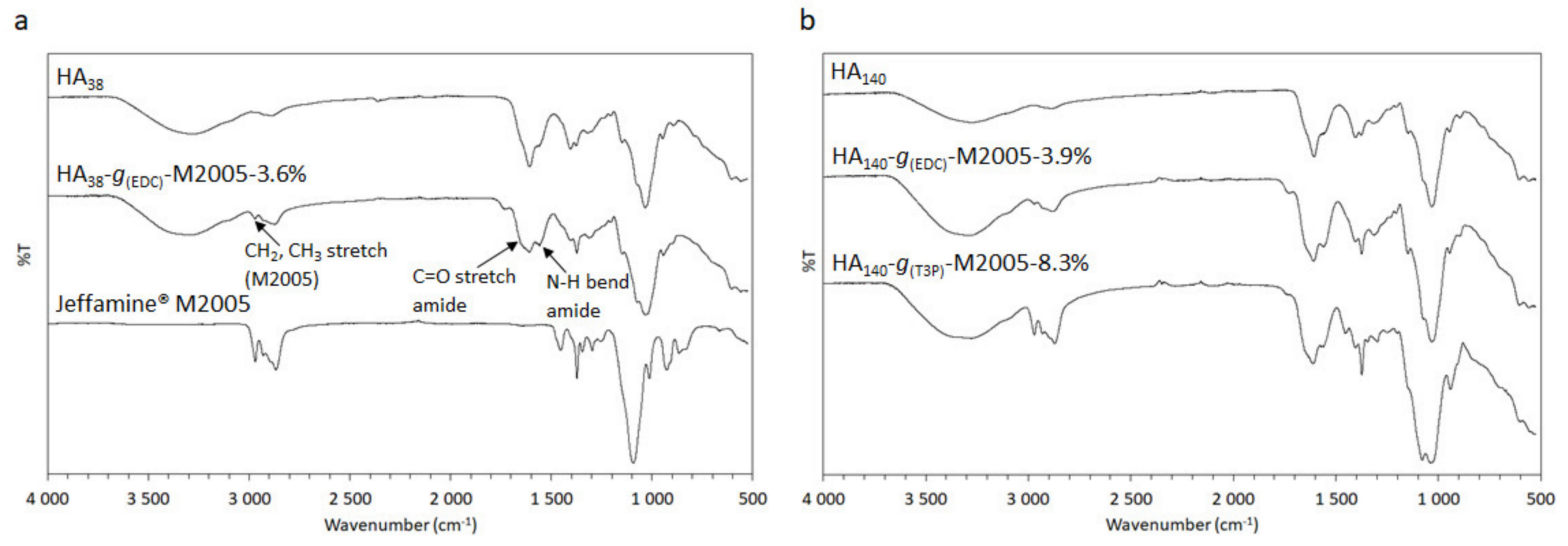
(**a**) FTIR spectrum of HA_38_ (**top**), HA_38_-g_(EDC)_-M2005-3.6% (**middle**), and M2005 (**bottom**) and (**b**) HA_140_ (**top**), HA_140_-g_(EDC)_-M2005-3.9% (**middle**), and HA_140_-g_(T3P)_-M2005-8.3% (**bottom**).

The successful grafting could be qualitatively assessed through FTIR, with bands corresponding to M2005 appearing at 2875 cm^−1^ and 2970 cm^−1^ (corresponding to the stretching of, respectively, C-H_2_ and C-H_3_ from the etheroxide backbone) and at 1375 cm^−1^ (corresponding to the bending of C-H_2_ from the etheroxide backbone). Moreover, a slight increase of the band at 1565 cm^−1^ (corresponding to N-H bend from amide) can be observed, suggesting the formation of additional amide bonds (Figure 1) on the structure of HA.

**Figure 2:**
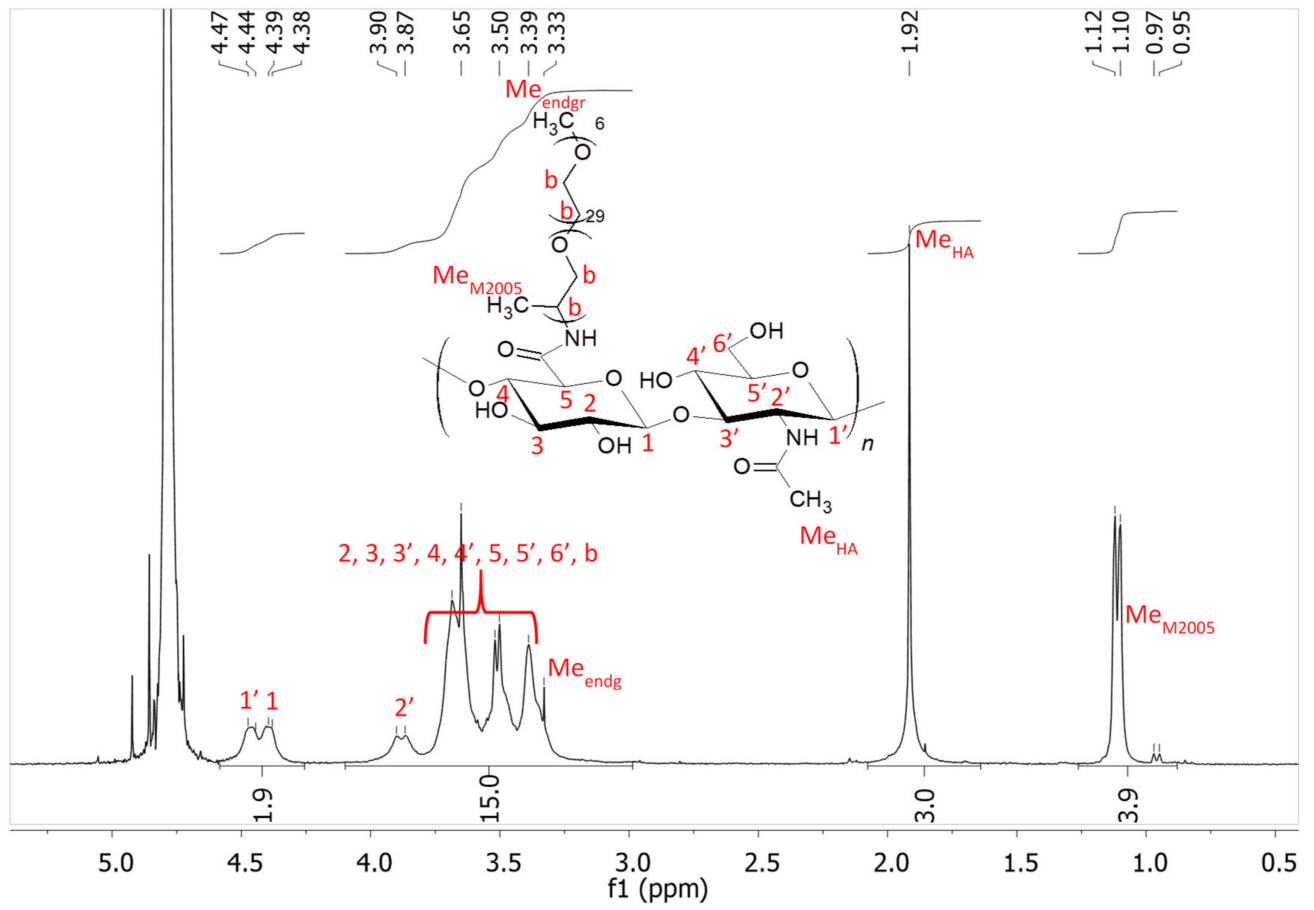
^1^H NMR spectrum of HA_1200_-g_(EDC)_-M2005-4.5% in D_2_O.

The grafting reaction efficiency was low in all the cases, none of the samples having a degree of substitution of more than 5 % (Figure 2). 

Nonetheless, some differences between the different HA materials can be spotted (Table 1): the grafting reaction was indeed slightly less efficient for HA, which had the highest starting molar mass with a DS of 2.7 %, while the other two batches had a grafting degree between 3.5 % and 4 %. Moreover, the reaction led to some polymer degradation for HA_1200_, with the molar mass values becoming around half the starting values, while HA_38_ and HA_140_ were almost unaffected by the reaction (Figure S2). This could be due to the dialysis step, which was partly performed against 0.1 M NaOH to remove EDC from the material (which will induce HA degradation as it is well known that this polysaccharide is prone to β-elimination when exposed to alkaline solution) [26]. The grafting degree could be improved for HA_1200_ by using an excess of M2005 compared to HA. When going for a ratio of M2005 chains compared to HA units of 5:1 instead of 1:1, the resulting DS was found to be 4.5%.

### 2.2. Synthesis of HA-g_(T3P)_-M2005 in Polar Aprotic Solvent

**Figure 3:**
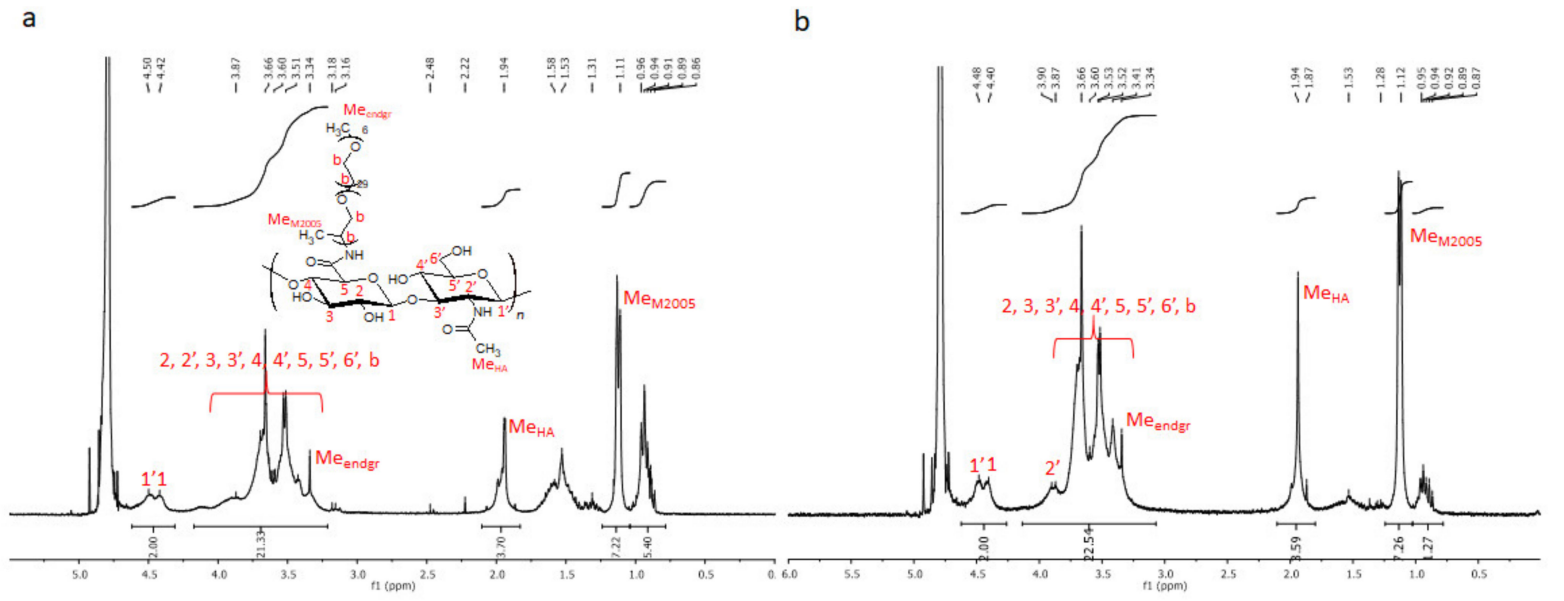
^1^H NMR spectrum of (**a**) HA_38_-*g*_(T3P)_-M2005-8.3% and (**b**) HA_140_-*g*_(T3P)_-M2005-8.3% in D_2_O.

The reaction was performed in DMF with T3P^®^ as the coupling reagent at room temperature. Grafting degrees of 8.3% were reached after functionalization of HA_38_ and HA_140_ (Figure 3). The resulting functionalized HAs using this route were coded as follows: HA_Mn(kg.mol_^−1^)-*g*_(T3P))_-M2005-DS%. The higher degree of substitution could be qualitatively assessed through FTIR, as the bands corresponding to M2005 were more pronounced (Figure 1). However, it should be noted HA_140_-*g*_(T3P)_-M2005-8.3% prepared in this way displayed hydrogel behaviour at a 60 g.L^−1^ solution in water, even at low temperature, and was looked like a colloidal suspension at 30 g.L^−1^,which implies the formation of cross-links. Trying to reduce the amount of coupling reagent for functionalizing HA_140_ and HA_1200_ (resulting in samples HA_140_-*g*_(T3P)_-M2005-5.5% and HA_1200_-*g*_(T3P)_-M2005-3.7%) also led to hydrogel behaviour in water in the case of HA_140_ and to a colloidal suspension in the case of HA_1200_. 

The synthesis in DMF using T3P^®^ as the coupling reagent proved therefore to be more efficient than the one performed in water, with DS above 5% being reachable. However, it is linked to unexpected behaviour in the case of both HAs with the highest starting molar masses, HA_140_ and HA_1200_, which is probably linked to cross-linking formation. The impact on the material behaviour will be investigated in the next section.

### 2.3. Physicochemical Properties of HA-g-M2005

#### 2.3.1. Conformational Properties Extracted from SEC/MALS

##### Reaction with EDC/NHS in Water

The physicochemical parameters of the various HA-*g*-M2005 were determined through SEC/MALS and rheology. As shown in Table 2, at first glance, the reaction performed in water with EDC/NHS as a coupling reagent did not impact the molar mass of the material obtained from HA_38_ and HA_140_, as the grafting degree is rather low (between 3.5% and 4%). In fact, when the results are looked at closely, a slight material degradation can be evidenced: the degree of polymerisation (DP_n_) of the chain has been determined according to Equation (S2), and it can be seen that the DP_n_ of functionalised HA_38k_ and HA_140k_ is equal to 84 % of its initial value. Moreover, the intrinsic viscosity [η] is found to slightly decrease in both cases, going from 150 mL.g^−1^ to 120 mL.g^−1^ in the case of HA_38_ and from 410 mL.g^−1^ to 390 mL.g^−1^ in the case of HA_140_. This can be attributed both to the grafting of M2005 to HA, leading to the disruption of the many intermolecular H-bonds between HA chains and to the slight HA-*g*-M2005 degradation [10,13,19]. Conversely, the “a” parameter from the Mark Houwink equation does not change much with the grafting reaction, being always around 0.7, suggesting that the resulting materials all display a random coil conformation in good solvent (they were not determined in the case of HA_1200_ due to the challenging analysis). In the case of HA_1200_, as described earlier, the reaction leads to material degradation, with resulting molar masses being half the initial values. This degradation is further indicated by a drop of both the hydrodynamic radius, R_h_, and the gyration radius, R_g_ (Table 2). However, it can be seen HA_1200_-*g*_(EDC)_-M2005-2.7% and HA_1200_-*g*_(EDC)_-M2005-4.5% have very similar molar masses, with M_n_ values of respectively 430,000 g.mol^−1^ and 540,000 g.mol^−1^, thus making the comparison of their properties with the different grafting degree possible. It can indeed be seen that despite having the highest molar mass, HA_1200_-*g*_(EDC)_-M2005-4.5% has a lower [η] and R_g_ compared to HA_1200_-*g*-M2005-2.7%, which can be expected due to the higher grafting degree leading to higher intermolecular interaction disruption. This result is in agreement with a previous study on the grafting of M2005 on carboxymethylpullulan [8].

##### Reaction with T3P^®^ in DMF

**Figure 4:**
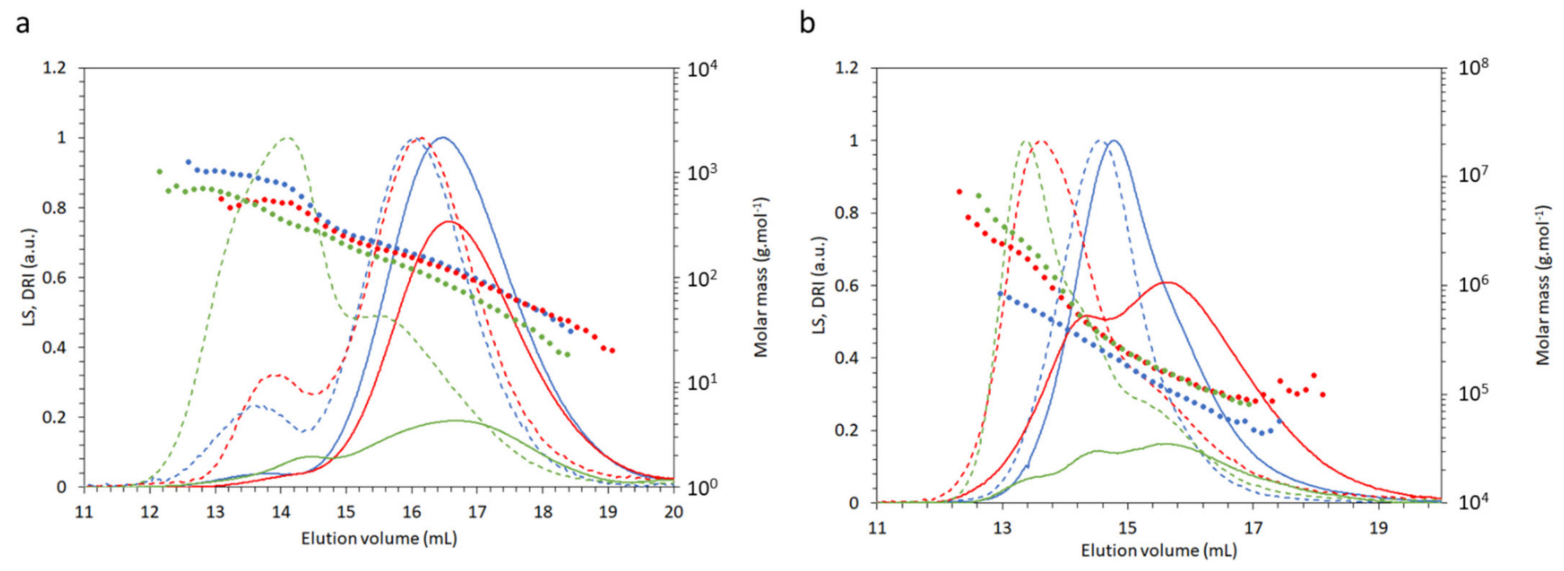
(**a**) Molar mass distribution of HA_38_ (blue), HA_38_-*g*_(EDC)_-M2005-3.6% (red), and HA_38_-*g*_(T3P)_-M2005-8.3% (green) (**b**) Molar mass distribution of HA_140_ (blue), HA_140_ after reticulation with T3P^®^ (red), and HA_140_-*g*_(T3P)_-M2005-8.3% (green); full lines: DRI; dotted lines: LS; full circles: molar mass distribution.

The chromatograms obtained for HA-*g*_(T3P)_-M2005 revealed a different behaviour than for HA-*g*_(EDC)_-M2005. First of all, while mass recovery after filtration as detected from DRI was high (>60 %) for almost all samples modified in Milli-Q water with EDC/NHS, it was below 50% for all samples modified with T3P^®^ in DMF, with HA_1200_-*g*_(T3P)_-3.7% being fully retained by the filter. Moreover, the molar masses were higher than the corresponding HA (e.g., HA_38_-*g*_(T3P)_-M2005-8.3% displayed respectively a M_n_ of 60,000 g.mol^−1^ (38,000 g.mol^−1^ for starting HA_38_) and a M_w_ of 250,000 g.mol^−1^ (64,000 g.mol^−1^ for starting HA_38_)) and the dispersities (Đ) were increased (4.18 for HA_38_-*g*_(T3P)_-M2005-8.3% versus 1.68 for HA_38_). In all cases, the LS signal main peak is shifting to the higher values (Figure 4a), indicating the formation of aggregates.

In order to investigate the impact of the coupling reagent T3P^®^, the coupling reaction was performed on HA_140_ as stated in the material section without adding Jeffamine^®^ M2005. The resulting material, not fully soluble in Milli-Q, displays the same molar mass values compared to functionalized HA_140_-*g*_(T3P)_-M2005, similar chromatograms (see Figure 4b) and also significant mass loss upon filtration with a mass recovery of only 41 %, all of which are hinting toward cross-linking of the sample. It is known coupling reagent that can trigger cross-linking of polysaccharide chains: 2-chloro-1-methylpyridinium iodide (CMPI) has already been used to cross-link HA of high molar masses [27] for instance. Trisodium trimetaphosphate, which has a chemical structure akin to T3P^®^, is also a well-known cross-linker of HA in water [28]. This side-reaction was still unexpected, as no cross-linking induced by T3P^®^ was observed on alginate of low molar mass (30,000 g.mol^−1^) by Schleeh et al., as evidenced by their SEC-MALS data [25]. Moreover, the cross-linking occurred in spite of the reaction conditions, where the starting concentration of HA was reduced with increasing initial molar mass (see Section 4.2.2). Furthermore, it is interesting to notice HA_140_ which was cross-linked with T3P^®^ without adding any Jeffamine^®^ M2005 displayed higher [η]_w_, Rh_w_ or Rg_w_ than HA_140_-*g*_(T3P)_-M2005 in 0.1 M LiNO_3_ solution, indicating the polymer functionalized with the polyetheramine is less swollen than the corresponding material which was only cross-linked with the coupling reagent. 

##### Rheological Properties and Temperature Induced Hydrogel Formation

**Figure 5:**
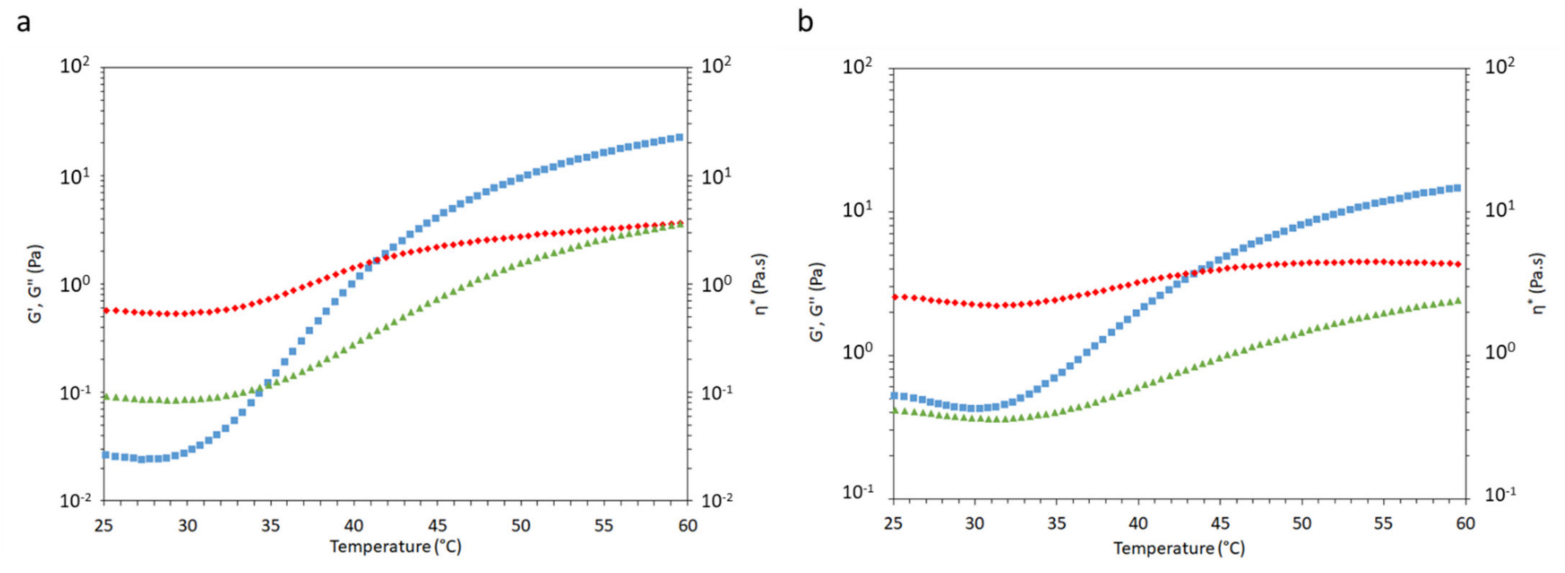
Rheological profile of (**a**) HA_1200_-*g*_(EDC)_-M2005-4.5% at 1 wt% in water and (**b**) HA_1200_-*g*_(EDC)_-M2005-2.7% at 1 wt% in water. Heating ramp: G′ (blue square), G″ (red rhombus), η* (green triangle) vs temperature; measurement in oscillation mode (parameters: shear stress: 0.1 Pa; frequency: 1 Hz; rate: 0.5 °C.min^−1^).

The thermal transition of the samples was assessed through rheological measurements in water. It was determined as the crossing between the storage modulus G′, corresponding to the fluid elastic behaviour, and the loss modulus G″, corresponding to the fluid viscous behaviour [8,29]. As the temperature rises, M2005-M2005 interactions are favoured over M2005-water ones, thus creating non-covalent cross-linking between HA-*g*-M2005 chains through the grafted moieties. The material will therefore go from an initial state at the lower temperature where G″ is over G′, indicating the viscous behaviour of the sample is predominant to a state where G′ is over G″ and a gel-like behaviour, the structure being able to recover its initial shape after a slight deformation. However, the transition will only be observed if the cross-linking is made through an intermolecular process, which requires the sample solution to be above its C* (Figure S3). The solutions were therefore prepared at a concentration 12.5 times above the C* of starting HA, and the results are displayed in Figure 5 and Table 3.

##### Rheological Properties of HA-g_(EDC)_-M2005

The temperature ramps were applied from 25 °C to 60 °C. The rheological response of HA_1200_-*g*_(EDC)_-M2005-4.5%, prepared as a solution in Milli-Q water at 1 wt%, was taken as an example in Figure 5a. In a first step, at between 25°C and 29°C, the complex viscosity η* is slightly decreasing, as it would be the case with non-thermo-sensitive material, even though the temperature is higher than the LCST of M2005. Although M2005 intermolecular associations will be promoted, as they are grafted to a very hydrophilic HA backbone, their temperature-induced hydrophobicity is not yet strong enough for triggering network formation. However, when the temperature reaches 28–29 °C, as cross-links are starting to enfold, G′, G″, and η* start to increase as the temperature rises. The crossing of G′ and G″ was reached at 41.5 °C, and all parameters were still rising as the temperature of 60 °C was reached, indicating the cross-linking formation was still strengthening. However, the measurement was stopped at this temperature, as water would start to evaporate above, even when using a solvent trap. 

As can be seen from Table 3 and Figure 5a,b, the grafting degrees did not impact the transition temperature for HA-*g*_(EDC)_-M2005 much, as observed when comparing HA_1200_-*g*_(EDC)_-M2005-2.7% and HA_1200_-*g*_(EDC)_-M2005-4.5%, their transition temperature being respectively found at 43.7 °C and 41.5 °C. This is most probably because the achievable DS are still too close to each other. All the HA-*g*_(EDC)_-M2005 obtained have therefore sufficiently close DS to be compared according to the starting HA batch. It is clear from Table 3 that the transition temperature increases with decreasing starting HA molar mass, with HA_38_-*g*_(EDC)_-M2005-3.6% displaying the highest value, just above 60 °C, despite the chains being in an entangled state.

We also compared the G′ value at 60 °C, which gives an indication of the gel strength, as according to the Hooke’s law, the higher it is, the higher the required strength to strain the material to a set value will be. Among all HA-*g*_(EDC)_-M2005 samples, the material with the highest G′ at 60 °C is HA_140_-*g*_(EDC)_-M2005-3.9% with 296 Pa. The behaviour is actually difficult to predict, as it is the consequence of several parameters, such as the material conformation in solution, the entanglement and the viscosity. However, it should be noted that a high variation of G′ during the thermal transition, corresponding to a component going from a very fluid state to a highly rigid one, is useful when devising materials for injection applications. The transition reversibility was also studied by applying a cooling ramp right after the heating ramp with the same general parameters, and all materials were giving a similar response as during the heating ramp, with only slight shifts of the temperature transition (<3 °C) (Figure S4).

##### Rheological Properties of HA-g_(T3P)_-M2005

**Figure 6:**
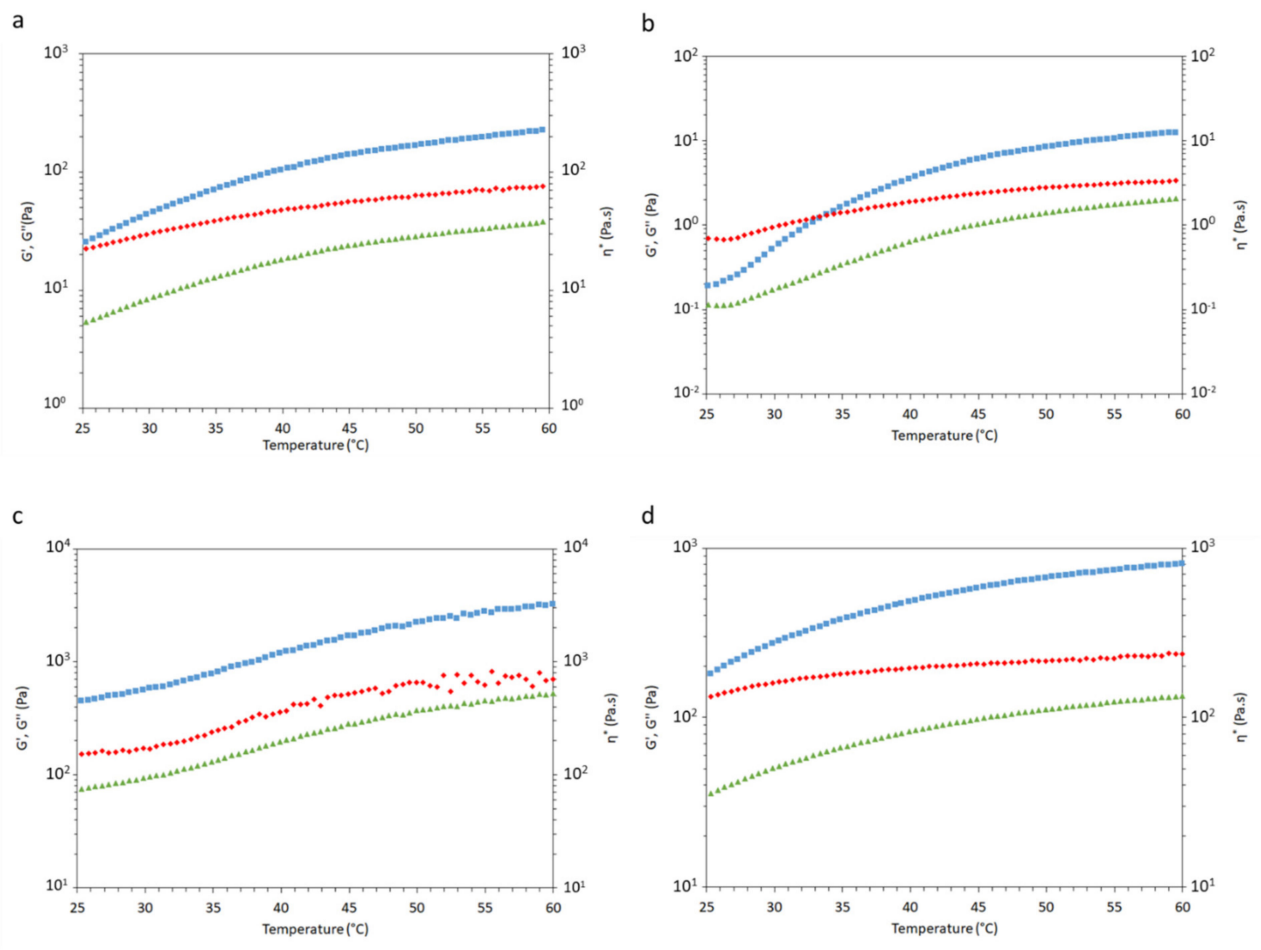
Rheological profile of (**a**) HA_38_-*g*_(T3P)_-M2005-8.3% at 11 wt% in water (**b**) HA_38_-*g*_(T3P)_-M2005-8.3% at 3 wt% in water (**c**) HA_140_-*g*_(T3P)_-M2005-8.3% at 6 wt% in water and (**d**) HA_140_-*g*_(T3P)_-M2005-5.5% at 6 wt% in water. Heating ramp: G′ (square), G″ (rhombus), η* (triangle) vs. temperature; measurement in oscillation mode (parameters shear stress: 0.02 Pa (**a**,**b**), 0.1 Pa (**c**) or 0.5 Pa (**d**); frequency: 1 Hz; heating rate: 0.5 °C.min^−1^).

In the case of HA-*g*_(T3P)_-M2005, only HA_38_-*g*_(T3P)_-M2005-8.3% was soluble in water, with HA_140_ derivatives behaving like a gel and HA_1200_ derivatives behaving like a colloidal suspension. Focusing first on HA_38_-*g*_(T3P)_-M2005-8.3%, the material was already displaying a G′ value above the G″ when working under the same conditions as HA_38_-*g*_(EDC)_-M2005-3.6% (11wt% solution in Milli-Q water) (Figure 6a), with all moduli values increasing over the whole temperature range. In order to see a transition, another solution was prepared with a concentration of only 3 wt% (Figure 6b). The transition was seen at 33.7 °C only, the lowest observed during this study, with G′ values at 25 °C and 60 °C similar to the ones of HA_1200_-*g*-M2005-2.7% at 1 wt%. In the case of the HA_140_-*g*_(T3P)_-M2005 samples, both of them were displaying a hydrogel behaviour at a concentration of 60 g.L^−1^ (G′ > G″ over the whole range) (Figure 6c,d) and a colloidal suspension behaviour at 20 g.L^−1^ (Figure S5). Nonetheless, the materials were still displaying thermo-responsive behaviour, with G′ and G″ values both increasing during the heating ramp (Table 3). The magnitude of G′ increase between 25 °C and 60 °C was between 4.6 and 9.2 (Table 3), which is far below the values found for materials starting with a predominant viscous behaviour (G″ > G′), with G′_60°C_ being at least 60 times higher than G′_25 °C_. However, the strongest gels were obtained in this way, with G′_60 °C_ values being all above 230 Pa. The functionalization with T3P^®^ is therefore more suited to obtain chemical hydrogels which are strengthening their network through the rise of temperature, which induces the establishment of additional reversible physical cross-links. In case a material displaying a thermo-responsive hydrogel transition behaviour is needed, it is better to work with low molecular weight HA as HA_38_-*g*_(T3P)_-M2005-8.3% displayed an interesting G′/G″ crossing at 33.7 °C.

## 3. Conclusions

Jeffamine^®^ M2005 was grafted to hyaluronic acids of various molar mass using two different synthesis approaches with varying grafting degrees. The route using water as the solvent and a combination of EDC and NHS as coupling reagents did not allow to reach a degree of substitution of more than 5%. However, it was sufficient to induce a transition to a gel-like structure around 40 °C, with functionalized HA_1200_ at only 1 wt% concentration. On the other hand, with this range of grafting degree, HA_38_-*g*_(EDC)_-M2005 and HA_140_-*g*_(EDC)_-M2005 did not reach a gel-like structure with a reasonable concentration of the material in water, the transition happening at more than 50 °C for already high concentrations of respectively 11 wt% and 6 wt%. In general, the protocol in water is straightforward to use and compatible with green chemistry principles, with the only drawback arising from the need to carefully purify the resulting material with several washings in acetone, as dialysis was not efficient to fully remove unreacted M2005. Moreover, although HA_38_ and HA_140_ remained intact from the reaction, HA_1200_ was slightly degraded, avoiding degradation would most probably lead to material with even better sol-gel transition properties.

In the approach that used DMF as the solvent and T3P^®^ as the coupling reagent, higher grafting degrees of around 8% were achieved compared to the protocol in water. However, poorly soluble materials were obtained for HA_140_ and HA_1200_ due to cross-linking formation, even when reducing the amount of coupling reagent. Still, the materials were also displaying thermo-responsive behaviour because of the formation of additional physical cross-links upon a chemical hydrogel network. Only HA_38_ led to soluble samples, which displayed improved properties compared to the approach with water, with a high tendency to aggregate and a transition to a gel-like behaviour at only 33.7 °C with a concentration of only 3 wt% in water. This material would be promising for devising injectable hydrogels for biomedical applications [30].

## 4. Materials and Methods

### 4.1. Materials

Hyaluronic acid sodium salt from *Streptococcus equi* (M_n_: 1,200,000 g.mol^−1^, abbreviated as HA_1200_) was purchased from Sigma-Aldrich (Saint-Quentin-Fallavier, France), and two sodium hyaluronate batches with lower molar masses were kindly provided by Givaudan France SAS (Pomacle, France): BASHYAL with M_n_: 140,000 g.mol^−1^ (HA_140_) and RENOVHYAL with M_n_: 38,000 g.mol^−1^ (HA_38_) (absolute molar masses were determined through SEC/MALS measurements as detailed in Section 4.3.3). Jeffamine^®^ was kindly provided by Huntsman. EDC, NHS, sodium phosphate monobasic monohydrate (NaH_2_PO_4_.H_2_O), and tetrabutylammonium hydroxide 54–56 wt% (TBAOH) were purchased from Sigma-Aldrich (Saint-Quentin-Fallavier, France). Propylphosphonic anhydride (T3P^®^) solution 50 wt% in DMF was purchased from Acros Organics (Schwert, Germany). Anhydrous DMF, sodium chloride, sodium hydroxide, hydrochloric acid, sodium hydrogenophosphate (Na_2_HPO_4_), and amberlite ion exchange resin (IRN77, cationic type, H^+^) were purchased from VWR (Fontenay-sous-Bois, France). Triethylamine (TEA) was purchased from Fluka (United Kingdom) and sodium phosphate dibasic dihydrate (Na_2_HPO_4_.2H_2_O) was purchased from Merck (Rahway, NJ, USA). Water was purified with the Milli-Q water reagent system (Millipore, Burlington, MA, USA). 

### 4.2. Synthesis of HA-g-M2005 

#### 4.2.1. Synthesis in Water with EDC/NHS

The reaction was adapted from Dulong et al. [8] and typically done following the procedure described with eventual adjustments (see Scheme 1 for the mechanism). HA (500 mg, 1.25 mmol) was dissolved overnight in 87.5 mL Milli-Q water. At the same time, M2005 (2.47 g, 1.25 mmol) was dissolved overnight in 9 mL Milli-Q water at 4 °C, as its LCST is 14 °C. The day after, the HA solution was placed at 4 °C while the pH of the M2005 solution was adjusted to 4.7 using both 1 M and 0.1 M HCl and NaOH solutions. As the pH adjustment of the M2005 solution was performed at room temperature, the solution sometimes precipitated due to the increasing temperature; it was therefore placed back at 4 °C for a while to dissolve again, this step being more straightforward due to the presence of positively charged ammonium induced by the pH decrease. The M2005 solution was then added to the HA solution (5 mL Milli-Q water were used for rinsing), and the pH was once more adjusted to 4.7 M using HCl and NaOH, as previously described, with the resulting mixture placed back at 4 °C immediately afterwards. An EDC/NHS solution was then prepared with 238.8mg EDC (1.25 mmol) and 5.7 mg (0.05 mmol) NHS dissolved in 2.5 mL water (the NHS should remain in catalytic quantities in order for the reaction not to lose its efficiency as stated by Dulong et al) [8]. It was then added to the HA/M2005 solution to start the reaction, which was left to proceed for one day at 4 °C. The day after, the reaction mixture was placed in a dialysis membrane (cut off: 12−14 kDa for HA_140_ and HA_1200_; 3.5kDa for HA_38_) and several cycles of dialysis were performed: one hour against 0.1 M NaOH, followed by neutralization against water for two days (changing water several times within each day); one hour against 0.1 M HCl, followed by neutralization against water for two days (changing water several times within each day); one hour against 0.1 M NaOH, to convert back the carboxylic acid moieties to sodium carboxylate, followed by neutralization against water for two days, then two days against a mixture of EtOH/H_2_O (ratios: 33/67, *v*/*v*), and finally, against water until conductivity was as close to Milli-Q water as possible. In order to completely remove the unreacted Jeffamine^®^ M2005, the material was washed several times in acetone until no variation of the ^1^H NMR peaks corresponding to M2005 was observed (Figure S1). 

#### 4.2.2. Synthesis of HA-g-M2005 in Organic Solvent

- Conversion of sodium hyaluronate to tetrabutylammonium hyaluronate: 5 g of HA were dissolved overnight in 830 mL at room temperature. Then the cation exchange resin (H^+^) (45 g) was introduced in the solution, which was left to stir overnight. The resin was subsequently filtered off the solution through a sintered glass filter, whose porosity was adjusted depending on the viscosity of the HA solution. The obtained filtered hyaluronic acid solution was then neutralized with TBAOH for getting tetrabutylammonium hyaluronate TBA-HA as follows: Tetrabutylammonium hydroxide (TBAOH) 54–56 wt% in water was diluted fivefold with water prior to the reaction. It was subsequently added drop-wise to the previously prepared hyaluronic acid solution until the pH reached 8. The solution was then freeze-dried and the resulting solid kept in a freezer until further use.
- M2005 grafting on TBA-HA in DMF with T3P^®^*:* The grafting on reaction was adapted from Schleeh et al. [25], with an example given as follows: 2.5 g TBA-HA (4.03 mmol, from batch HA_38_) were dissolved in 30 mL anhydrous DMF at room temperature in a three-neck flask which was thereafter put under nitrogen. Then, 4.7 mL of T3P^®^ (50 wt% in DMF) were added to the mixture, which was left to stir for one hour at room temperature. Meanwhile, 15.96 g M2005 were dissolved in 15 mL anhydrous DMF. After TBA-HA was activated by T3P^®^ for one hour, 568 µL of TEA were added to the solution, immediately followed with the M2005 solution (the flask was rinsed with 2.5 mL DMF). The final TBA-HA concentration was 50 g.L^−1^, which was respectively adjusted to 10 g.L^−1^ for HA_140_ and to 6 g.L^−1^ for HA_1200_ by increasing the amount of DMF used to dissolve TBA-HA. The reaction was left overnight at room temperature. The day after, 25 mL of a 2.5 M solution of NaCl were added drop-wise to the reaction vessel, which was subsequently left to stir for 30min before being poured in 250 mL acetone to precipitate the modified hyaluronic acid. The material was then recovered through filtration, dissolved in 350 mL 0.1 M phosphate buffer (pH: 7.5) and then dialyzed against water until the resulting conductivity of water was found to be as close as Milli-Q as possible, indicating removal of the impurities; this was followed eventually by freeze-drying of the material. In order to completely remove the unreacted M2005, the material was washed several times in acetone until no more variation of the ^1^H NMR peaks corresponding to M2005 was observed.

### 4.3. Characterisation Methods

#### 4.3.1. Infrared Spectroscopy

Infrared spectra were acquired on a spectrometer Nicolet IS50 FT-IR (Thermo Scientific, Waltham, MA, USA). The samples were analysed with the OMNIC 8.0 software by transmission from 500 to 4000 cm^−1^ (32 scans with resolution 2). 

#### 4.3.2. ^1^NMR Spectroscopy

^1^H NMR spectra were acquired on a Bruker Avance 300MHz spectrometer (Billerica, MA, USA). The PMeOx homopolymer samples were prepared in deuterated chloroform CDCl_3_, while HA derivatives were prepared in deuterium oxide D_2_O containing 0.125 M sodium deuteroxide NaOD [11].

The M2005 grafting degree (DS) on HA was evaluated by ^1^H NMR using either Equation (1) for HA-*g*_(EDC)_-M2005 (using HA methyl protons as a reference) or Equation (2) for HA-*g*_(T3P)_-M2005 (using HA anomeric protons as a reference in case there were peaks overlapping with HA methyl protons).

(1) $$DS=\frac{I_{Me_{M2005}}}{\left(3\times 29\right)}\times \frac{3}{I_{Me_{HA}}}$$

where $I_{Me_{M2005}}$ corresponds to the integration of the duet of the methyl group from the propylene oxide units of the M2005 moieties found at about 1.10 ppm (the small duet at 0.95 ppm was also included in the integration, as it corresponds to the propylene oxide unit in α of the terminal nitrogen of M2005). $I_{Me_{HA}}$ corresponds to the integration of the methyl group from the *N*-acetylglucosamine unit (Figure 4).

(2) $$DS=\frac{I_{Me_{M2005}}}{\left(3\times 29\right)}\times \frac{2}{I_{H_{anom}}}$$

where $I_{H_{anom}}$ corresponds to the integration number of the HA two anomeric protons were found at 4.47–3.38 ppm. The results of the various experiments have been combined in Table 1.

#### 4.3.3. SEC/MALS/DRI/Viscometer

The absolute average molar mass and molar mass distribution of the polymers were determined at 25 °C on a system coupling on-line a size-exclusion chromatograph (SEC), a multi-angle light scattering system (MALS), a viscometer and a differential refractive index detector (DRI). The carrier was a 0.1 M LiNO_3_ solution which was filtered through a 0.1 µm filter unit (Millipore, Burlington, MA, USA), degassed on-line (DGU-20A3, Shimadzu, Japan), and eluted at a 0.5 mL.min^−1^ flow-rate (LC10Ai Shimadzu, Kyoto, Japan). The solutions to analyse were prepared in 0.1M LiNO_3_ at a concentration of respectively 0.5 g.L^−1^ (HA_1200_ based materials) and 1 g.L^−1^ (HA_140_ and HA_38_ based materials) and were passed through a 0.45 µm filter prior to injection which was performed on 100 µL of the solution with an automatic injector (SIL-20A Shimadzu, Kyoto, Japan). The SEC line consisted of an OHPAK SB-G guard column for protection and two OHPAK SB 806 and 804 HQ columns (Shodex Showa Denko K.K., Tokyo, Japan) in series. The column packing was a polyhydroxymethylmethacrylate gel. The MALS detector was a DAWN Heleos-II (Wyatt Technology Inc., Goleta, CA, USA) fitted with a 50 µl K5 cell and 18 photodiodes (normalized relative to the 90° detector using bovine serum albumin). The viscometer was a ViscoStar II (Wyatt Technology Inc., Goleta, CA, USA). The collected data were analysed using the Astra 6.1.7.16 software package (Wyatt Technology Inc, Goleta, CA, USA). Molar masses were obtained with the Zimm order 1 method. The concentration of each eluted fraction was determined with DRI (RID 10A Shimadzu, Kyoto, Japan) according to the known values of d*n*/dC (0.150 mL.g^−1^ for HA and derivatives).

#### 4.3.4. Rheology

Rheological measurements were performed on a Discovery Hybrid Rheometer HR-2 from TA Instruments (New Castle, DE, USA). Samples were prepared in Milli-Q water as a solvent at a concentration 12.5 times higher than starting HA overlapping concentration C*. Geometries used for measurements were either double concentric cylinders (aluminium—gap: 500 µm) or cone-plate (aluminium—gap: 50 µm). Oscillation measurement were mostly performed with a set frequency of 1 Hz (for measurement other than frequency sweep) and a set stress of 0.1 Pa (for measurement other than stress sweep). Temperature ramps were performed from 25 °C to 60 °C (and vice-versa) with a heating/cooling rate of 0.5 °C/min. A solvent trap was used to prevent the evaporation of water at the highest used temperatures. The data were processed using the Trios software v4.1.1.33073 from TA Instruments (New Castle, DE, USA).

## Figures and Tables

**Table 1:** Reaction conditions and resulting grafting degrees and molar mass of the different HA-g-M2005. EDC/NHS were used with a ratio of 1/0.04 compared to Na-HA units.

Sample	n_M2005_/n_HA_	n_T3P_/n _TBA-HA_	n_TEA/_n_TBA-HA_	DS
HA_38_-*g*_(EDC)_-M2005-3.6%	1	-	-	3.6 % ^a^
HA_38_-*g*_(T3P)_-M2005-8.3%	2	2	1	8.3 % ^b^
HA_140_-*g*_(EDC)_-M2005-3.9%	1	-	-	3.9 % ^a^
HA_140_-*g*_(T3P)_-M2005-5.5%	2	0.3	0.3	5.5 % ^b^
HA_140_-*g*_(T3P)_-M2005-8.3%	2	2	1	8.3 % ^b^
HA_1200_-*g*_(EDC)_-M2005-2.7%	1	-	-	2.7 % ^a^
HA_1200_-*g*_(T3P)_-M2005-3.7%	2	0.3	0.3	3.7 % ^b^
HA_1200_-*g*_(EDC)_-M2005-4.5%	5	-	-	4.5 % ^a^

^a^ DS is calculated according to Equation (1); ^b^ DS is calculated according to Equation (2).

**Table 2:** Molar mass analysis and macromolecular data (intrinsic viscosity [η], hydrodynamic radius (Rh), gyration radius (Rg), and Mark–Houwink parameters (a and K)) of the obtained samples through SEC/MALS (at 25°C, in LiNO_3_ 0.1 M).

Sample	M_n_ (g.mol^−1^)	M_w_ (g.mol^−1^)	Đ	DP_n_	Mass Recovery	[η]_w_ (mL.g^−1^)	Rh_w_ (nm)	Rg_w_ (nm)	a (Mark Houwink)
HA_38_	38,000	64,000	1.68	95	68%	150	11	- ^a^	0.76
HA_38_-*g*_(EDC)_-M2005-3.6%	38,000	63,000	1.64	80	61%	120	10	- ^a^	0.72
HA_38_-*g*_(T3P)_-M2005-8.3%	60,000	250,000	4.18	106	35%	140	14	37	0.79
HA_140_	140,000	210,000	1.50	349	63%	410	23	44	0.71
HA_140_ (T3P) ^b^	190,000	550,000	2.89	473	41%	450	30	68	0.72
HA_140_-*g*_(EDC)_-M2005-3.9%	140,000	190,000	1.39	294	77%	390	22	39	0.62
HA_140_-*g*_(T3P)_-M2005-5.5%	180,000	430,000	2.39	354	47%	320	25	46	0.76
HA_140_-*g*_(T3P)_-M2005-8.3%	200,000	540,000	2.76	355	21%	290	26	48	0.77
HA_1200_	1,200,000	1,500,000	1.25	2990	65%	1700	73	147	-
HA_1200_-*g*_(EDC)_-M2005-2.7%	430,000	500,000	1.15	947	78%	1300	46	102	-
HA_1200_-*g*_(T3P)_-M2005-3.7%	-	-	-	-	0%	-	-	-	-
HA_1200_-*g*_(EDC)_-M2005-4.5%	540,000	710,000	1.32	1105	49%	840	44	84	-

MHS coefficient (a) has been determined by integrating the most linear part of bilogarithmic representation of intrinsic viscosity vs. molar mass; SEC/MALS/VD/DRI data accuracy: M_n_ ± 9%; M_w_ ± 6%; Đ ± 10%; [η]_w_ ± 0.5%; Rh_w_ ± 2%; Rg_w_ ± 6%; ^a^ Out of the range of the MALS detector (isotropic diffusion for lower masses fractions); ^b^ Sample reacted with T3P^®^ only.

**Table 3:** Rheological properties of the different HA-*g*-M2005 samples.

Sample	C (wt%)	T_trans_ (°C)	G′ (Pa) (25°C)	G′ (Pa) (60°C)	$\frac{G\prime_{60{}^{\circ}C}}{G\prime_{25{}^{\circ}C}}$
HA_38_-*g*_(EDC)_-M2005-3.6%	11	>60 °C	0.1 Pa	78.4 Pa	784
HA_38_-*g*_(T3P)_-M2005-8.3%	11	<25 °C	25 Pa	230 Pa	9.2
HA_38_-*g*_(T3P)_-M2005-8.3%	3	33.7 °C	0.2 Pa	12.6 Pa	63
HA_140_-*g*_(EDC)_-M2005-3.9%	6	51.6 °C	1 Pa	296 Pa	296
HA_140_-*g*_(T3P)_-M2005-5.5%	6	-	176 Pa	810 Pa	4.6
HA_140_-*g*_(T3P)_-M2005-8.3%	6	-	435 Pa	3200 Pa	7.4
HA_1200_-*g*_(EDC)_-M2005-2.7%	1	43.7 °C	0.5 Pa	14.8 Pa	30
HA_1200_-*g*_(EDC)_-M2005-4.5%	1	41.5 °C	0.026 Pa	23 Pa	885

Temperature ramp measurements were performed with an oscillation frequency of 1 Hz. Summary of the properties of the different HA batches is available in Table S1.

## Supplementary Materials

The following are available online at https://www.mdpi.com/article/10.3390/gels7030088/s1. Reference [31] is cited in the Supplementary Materials. Table S1: Summary of the properties of the different HA batches, Figure S1:HA_1200_-*g*_(EDC)_-M2005-4.5% DS evolution with the number of washes in Acetone,, Figure S2: Molar mass distribution of (a) HA_1200_, HA_1200_-*g*_(EDC)_-M2005-4.5% and (b) HA_140_ and HA_140_-*g*_(EDC)_-M2005-3.9%, Figure S3: Dilute and semi-dilute domains of HA_1200_ in Milli-Q water as determined through low shear viscosity measurements, Figure S4: Rheological profile of HA_1200_-*g*_(EDC)_-M2005-4.5% at 1 wt% in water (with cooling ramp), and Figure S5: Rheological profile of HA_140_-*g*_(T3P)_-M2005-8.3% at 2 wt% in water (with cooling ramp).
