# Supplementary material for: Hyaluronic Acid Functionalization with Jeffamine® M2005: A Comparison of the Thermo-Responsiveness Properties of the Hydrogel Obtained through Two Different Synthesis Routes
Source: Gels. 2021 Jul 9;7(3):88. doi: 10.3390/gels7030088 (PMC8293199; doi:10.3390/gels7030088)
Supplement: Supplementary file 1 [file gels-07-00088-s001.zip › gels-1275853-supplementary.pdf]

Supplementary Materials

1. Synthesis of HA-g(EDC)-M2005 in water.

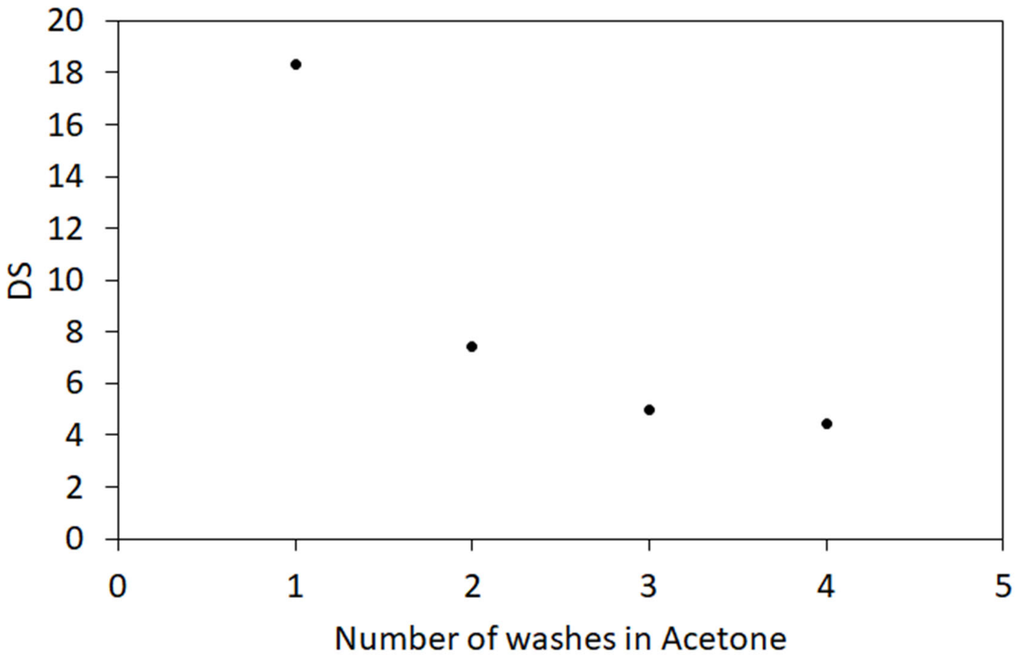

Figure S1. HA_{1200}-$g_{(EDC)}$-M2005-4.5% DS evolution with the number of washes in Acetone (DS was determined through ^{1}H NMR measurements).

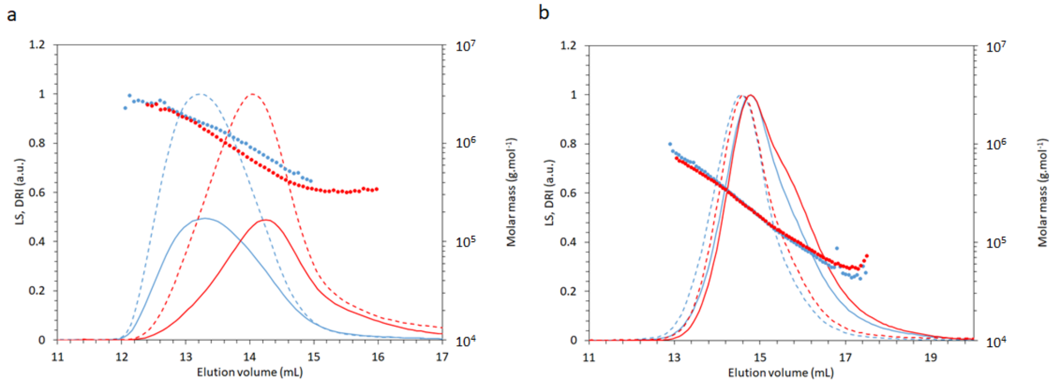

Figure S2. Molar mass distribution of a) HA1200 (blue), HA1200-g(EDC)-M2005-4.5% (red) and b) HA140 (blue), HA140-g(EDC)-M2005-3.9% (red); full lines: DRI; dotted lines: LS; full circles: molar mass distribution.

2. Characterisation of the starting HA batches

HA_{38}, HA_{140} and HA_{1200} dilute and semi-dilute domains were determined through low shear viscometry measurements using a Low Shear 400 Rheometer from Lamy Rheology (France). Samples were prepared in Milli-Q water. Measurements were performed in the Newtonian domain at low shear rate (≤ 5 s^{-1}, after having screened the starting solution at different shear rates) with a LS11 geometry. The HA solution was gradually diluted from the starting solution (whose concentration depended on the probed HA batch), so the dilution domains and the critical overlapping concentration C^{*} could subsequently be determined by plotting the evolution of the specific viscosity η_{spe} as a function of the HA mass concentration using a logarithmic scale. η_{spe} was determined using formulae. S1:

$$\eta_{spe} = \frac{\eta - \eta_0}{\eta_0} \quad (S1)$$

with η the measured viscosity of the solution and η_0 the viscosity of the solvent. The results of both SEC/MALS and C* measurements are summed up in table 1.

Table S1. Summary of the properties of the different HA batches.

Samples	M_n (g/mol) (SEC/MALS)	M_w (g/mol) (SEC/MALS)	C* (g/L, MQ) (Low Shear)
HA_{38}	38 000	64 000	8.5
HA_{140}	140 000	210 000	4.8
HA_{1200}	1 200 000	1 500 000	0.8

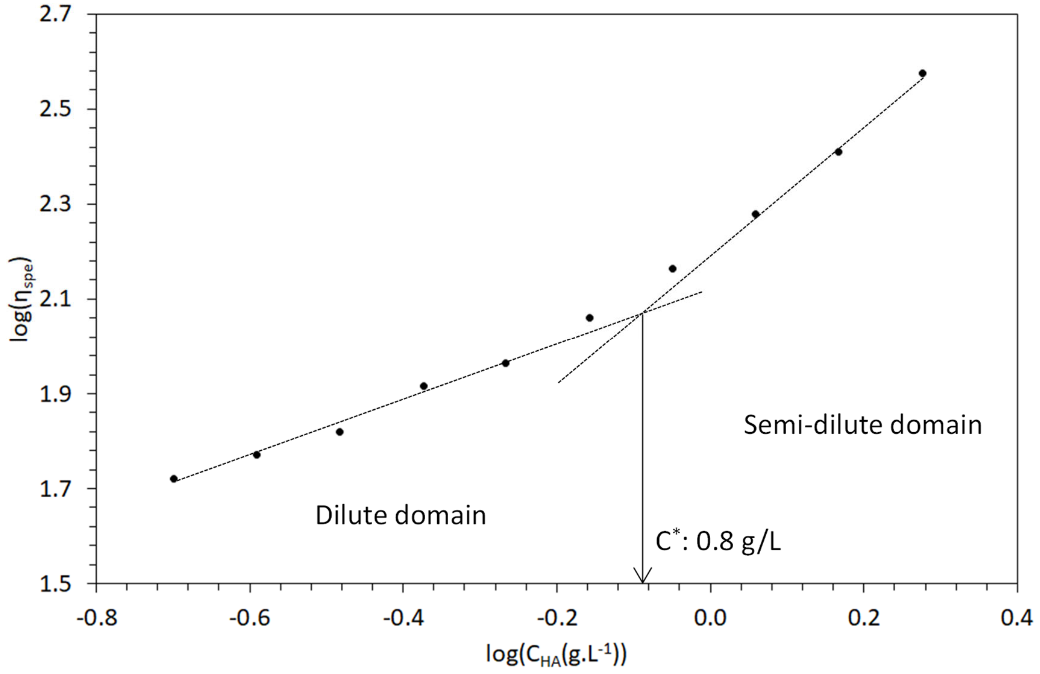

Figure S3. Dilute and semi-dilute domains of HA_{1200} in Milli-Q water as determined through low shear viscosity measurements. The crossing of the two linear domains is giving the C*.

The DP_n values of HA-*g*-M2005 samples reported in Table 2 were calculated using Eq. (S2):

$$DPn = \frac{Mn}{DS \times (M_{M2005} + Mu_{HA} - M_H) + (1 - DS) \times Mu_{NaHA}} \quad (S2)$$

Where M_n is the corresponding HA-*g*-M2005 average molar mass in number determined through SEC/MALS/VD/DRI measurements. M_{M2005} is the Jeffamine® M2005 molar mass, DS the grafting degree of M2005 on HA obtained from Eq. (1a) (HA-*g*(EDC)-M2005) or Eq. (1b) (HA-*g*(T3P)-M2005) with ^{1}H NMR spectroscopy (Fig. 3), M_{uHA} the molar mass of the acid form of HA disaccharide unit, M_H the molar mass of hydrogen and M_{uNaHA} the molar mass of the sodium form of HA disaccharide unit. Concerning M_{uNaHA}, for the sake of simplification, HA was assumed to be in its sodium salt form for this calculation. It is actually in equilibrium with the lithium salt form, also SEC/MALS/VD/DRI is only taking into account the fraction of condensed counter-ions [31]. Considering sodium or lithium salt will barely affect the calculated values, and more importantly, the observed tendencies will remain unaffected.

3. Rheological properties of HA-g-M2005

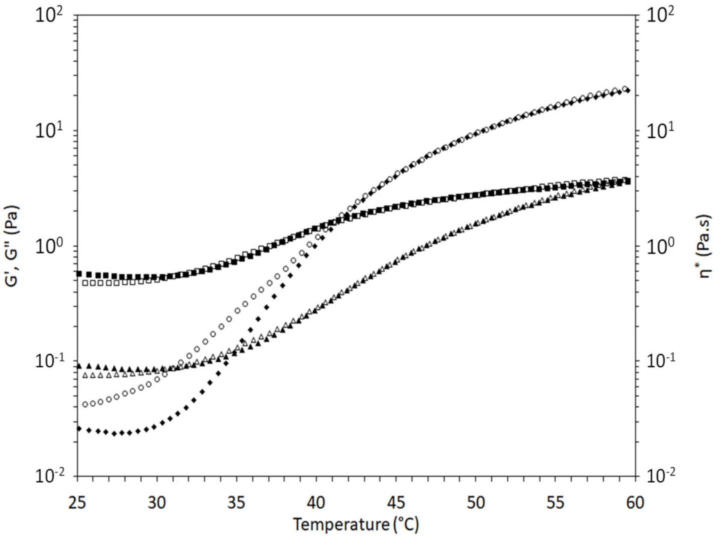

Figure S4. Rheological profile of HA$_{1200}$-*g*$_{(EDC)}$-M2005-4.5% at 1 wt% in water (with cooling ramp). Heating ramp: G′ (full square), G′′ (full rhombus), η* (full triangle) vs temperature; cooling ramp: G′ (empty square), G′′ (empty rhombus), η* (empty triangle) measurement in oscillation mode (parameters: shear stress: 0.1 Pa; frequency: 1 Hz; rate: 0.5 °C.min^{-1}).

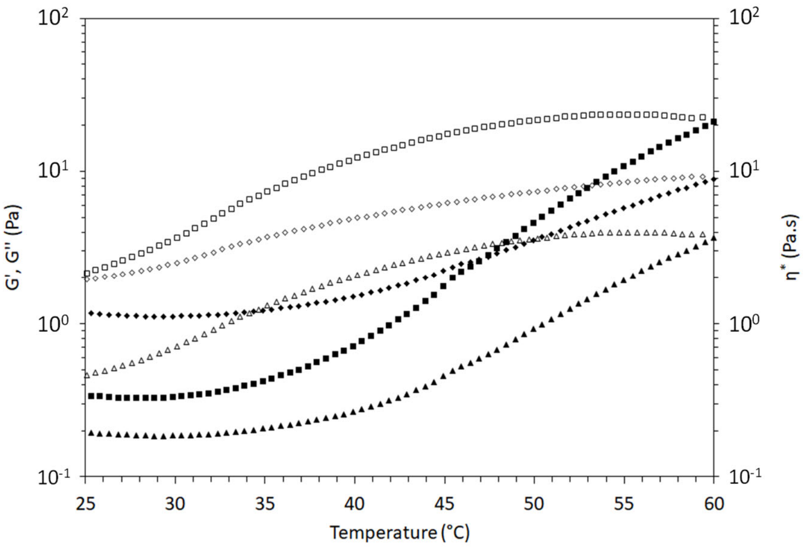

Figure S5. Rheological profile of HA$_{140}$-*g*$_{(T3P)}$-M2005-8.3% at 2 wt% in water (with cooling ramp). Heating ramp: G′ (full square), G′′ (full rhombus), η* (full triangle) vs temperature; cooling ramp: G′ (empty square), G′′ (empty rhombus), η* (empty triangle) measurement in oscillation mode (parameters: shear stress: 0.1 Pa; frequency: 1 Hz; rate: 0.5 °C.min^{-1}).
